# Enhanced Differential Evolution Algorithm with Local Search Based on Hadamard Matrix

**DOI:** 10.1155/2021/8930980

**Published:** 2021-10-29

**Authors:** Changshou Deng, Xiaogang Dong, Yucheng Tan, Hu Peng

**Affiliations:** ^1^School of Electronic Information Engineering, Jiujiang University, Jiujiang 332005, China; ^2^College of Information Management, Jiangxi University of Finance and Ecomomics, Nanchang 330013, China; ^3^College of Science, Jiujiang University, Jiujiang 332005, China

## Abstract

Differential evolution (DE) is a robust algorithm of global optimization which has been used for solving many of the real-world applications since it was proposed. However, binomial crossover does not allow for a sufficiently effective search in local space. DE's local search performance is therefore relatively poor. In particular, DE is applied to solve the complex optimization problem. In this case, inefficiency in local research seriously limits its overall performance. To overcome this disadvantage, this paper introduces a new local search scheme based on Hadamard matrix (HLS). The HLS improves the probability of finding the optimal solution through producing multiple offspring in the local space built by the target individual and its descendants. The HLS has been implemented in four classical DE algorithms and jDE, a variant of DE. The experiments are carried out on a set of widely used benchmark functions. For 20 benchmark problems, the four DE schemes using HLS have better results than the corresponding DE schemes, accounting for 80%, 75%, 65%, and 65% respectively. Also, the performance of jDE with HLS is better than that of jDE on 50% test problems. The experimental results and statistical analysis have revealed that HLS could effectively improve the overall performance of DE and jDE.

## 1. Introduction

Differential evolution (DE), which was proposed by Storn for solving Chebyshev inequality in 1995 [1], is a well-known numerical optimization algorithm. Due to its simple structure, limited number of parameters, an easy implementation, and outstanding optimization performance, DE has drawn great attention of many researchers and engineers since it was proposed. Over the past two decades, DE has been successfully applied to a variety of fields, such as computer vision [2], dynamic economic dispatch [3], engineering design [4], project scheduling [5], artificial neural networks [6], and complex problems inherent to magnetorheological fluids of interest to the automotive industry, in the framework of extended irreversible thermodynamics [7, 8]. Unlike other population-based evolutionary algorithms, the mutation operator in DE utilizes differential information between individuals in the current population. The mechanism gives DE an obvious edge over other evolutionary algorithms. The binomial crossover, however, only produces one offspring in the space constructed by the target individual and its descendant. Therefore, the trial individual is just only one case of many potential solutions, and other potential solutions are ignored. Hence, it is clear that DE's search of the subspace is insufficient. This clearly affected the overall performance of DE.

To fill this gap, we introduced a new scheme of local search based on the Hadamard matrix (HLS) for the sake of improving the overall performance of DE.

The remainder of this paper is organized as follows.  Section 2 introduces the basic elements of DE algorithm and reviews the related work.  Section 3 presents the details about the Hadamard local search. The experimental results are reported in Section 4, while Section 5 concludes this paper.

## 2. Background

### 2.1. Differential Evolution

DE algorithm consists of the following four steps.

#### 2.1.1. Initialization

Initialization is the first step of DE algorithm. It randomly generates a population which contains NP individuals in D-dimensional space. For the *i*th individual, the *j*th parameter was initialized by the following formula:

(1) $$\begin{array}{c} x_{ij}^{0}=L_{j}+\text{Rand}\left(0,1\right)\times \left(U_{j}-L_{j}\right), \end{array}$$

where Rand(0,1) is a uniformly distributed random number within the range [0,1] and *L*_*j*_ and U_*j*_ are the lower and upper bounds of the dimensional spaces, *i* ∈ [1, NP], *j* ∈ [1, *D*].

#### 2.1.2. Mutation Operator

Following initialization, the mutation operator was applied to each target individual *x*_*i*_^*G*^, thus generating a mutant  *v*_*i*_^*G*^. In view of the important implications that the mutation operators have on the ability of DE's global search, many researchers focus on the work of improving them. Six efficient and widely used operators [9] are listed below.

(i) DE/rand/1/:
  (2) $$\begin{array}{c} v_{i}^{G}=x_{r1}^{G}+F\times \left(x_{r2}^{G}-x_{r3}^{G}\right). \end{array}$$
(ii) DE/best/1:
  (3) $$\begin{array}{c} v_{i}^{G}=x_{best}^{G}+F\times \left(x_{r1}^{G}-x_{r2}^{G}\right). \end{array}$$
(iii) DE/rand/2:
  (4) $$\begin{array}{c} v_{i}^{G}=x_{r1}^{G}+F\times \left(x_{r2}^{G}-x_{r3}^{G}\right)+F\times \left(x_{r4}^{G}-x_{r5}^{G}\right). \end{array}$$
(iv) DE/best/2:
  (5) $$\begin{array}{c} v_{i}^{G}=x_{best}^{G}+F\times \left(x_{r1}^{G}-x_{r2}^{G}\right)+F\times \left(x_{r3}^{G}-x_{r4}^{G}\right). \end{array}$$
(v) DE/rand-to-best/1:
  (6) $$\begin{array}{c} v_{i}^{G}=x_{r1}^{G}+F\times \left(x_{best}^{G}-x_{r1}^{G}\right)+F\times \left(x_{r2}^{G}-x_{r3}^{G}\right). \end{array}$$
(vi) DE/current-to-best/1:

(7) $$\begin{array}{c} v_{i}^{G}=x_{i}^{G}+F\times \left(x_{best}^{G}-x_{i}^{G}\right)+F\times \left(x_{r1}^{G}-x_{r2}^{G}\right). \end{array}$$

#### 2.1.3. Crossover Operator

Crossover operator randomly combines the genes of the target and its mutant to produce a new offspring. The binomial crossover is the most commonly used method. It is expressed as follows:

(8) $$\begin{array}{c} u_{ij}^{G+1}=\left\{\begin{array}{cc} v_{ij}^{G}, & \text{if rand}\left(0,1\right)<\text{CR or }j=\text{rand}\left(i\right),\\ x_{ij}^{G}, & \text{otherwise,} \end{array}\right)\; \end{array}$$

where rand(0,1) is a uniform random number in [0,1] and CR  is the crossover probability.

#### 2.1.4. Selection Operator

The DE selection operator is a greedy strategy. From the target individual and its offspring, the one with the better fitness value will enter the next generation. The selection operator is shown in the following formula:

(9) $$\begin{array}{c} x_{i+1}^{G+1}=\left\{\begin{array}{cc} u_{i}^{G+1}, & \text{if }f\left(u_{i}^{G+1}\right)<f\left(x_{i}^{G}\right),\\ x_{i}^{G}, & \text{otherwise}. \end{array}\right) \end{array}$$

## 3. Related Work

Although DE has been successfully applied in many fields, it still needs to improve the performance of the algorithm in many other fields. Thus, several improved versions of DE were proposed by the researchers. These works can be divided into the following four categories.

### 3.1. Improvement of the Mutation Operator

Ramadas et al. proposed a ReDE algorithm introducing a revised mutation strategy for DE [10]. Mohamed and Almazyad et al. proposed an ANDE algorithm which introduced a new triangular mutation for DE [11]. Gong and Cai proposed a classification-based mutation strategy for DE [12], in which some of the parents are selected proportionally based on their classification in the current population. Peng et al. proposed an improvement in differential evolution, which was named RNDE. RNDE used a new mutation operator, DE/neighbor/1, to balance the exploration and exploitation ability of DE process [13].

### 3.2. The New Scheme of Self-Adapting Parameters

Brest et al. presented a new approach to the self-adaptive control parameter of DE [14]. Qin et al. proposed a self-adapting DE algorithm, in which the strategies for generating test vectors and their associated parameter values are progressively self-adapting, taking advantage of their previous experiences in the generation of promising solutions [15]. Zhu et al. proposed an adaptive population adaptation scheme (APTS) for DE to dynamically adjust the size of the population [16].

### 3.3. Hybrid DE

Wang et al. proposed an orthogonal crossover (OX) operator, which is based on orthogonal design and can make a systematic and rational search in a region defined by the parent solutions [17]. Rahnamayan proposed an opposition-based learning DE (ODE). ODE employs opposition-based learning (OBL) for population initialization and for generation jumping [18]. Sun et al. proposed a new algorithm, named DE/EDA [19], which combined DE with estimation of distribution algorithm. Peng et al. proposed a novel DE variant with commensal learning and uniform local search, named CUDE. The biggest contribution of CUDE is to enhance the local space search performance of DE by using uniform experimental design [20].

### 3.4. The New Methods of Local Search Strategy

Local search can effectively improve the performance of evolutionary algorithms. For example, fittest individual refinement (FIR) was proposed by Noman et al. [21]. In the FIR, the search space around the best individual is explored greedily in each generation. Later, two implementations (DEfirDE and DEfirSPX) of FIR were proposed. The results of the experiments show that both schemes speed up DE for a set of well-known test functions, especially for high dimensions, and they are better than the other two well-known variants of DE. A crossover-based adaptive local search (LS) operator was proposed to enhance the performance of the standard DE algorithm [22]. The new algorithm mainly improved the local search by adaptively adjusting the length of the search using a hill-climbing heuristic. Trigonometric local search (TLS) and interpolated local search (ILS) were proposed in [23]. Combining these two local search strategies, two new variants of DE algorithms (DETLS and DEILS) were implemented. The new scheme improved the performance of DE in terms of the quality of solution without compromising on the convergence rate. A restart differential evolution algorithm with local search mutation (RDEL) was proposed in [24]. In RDEL, a novel local mutation rule based on the positions of the best and the worst individuals among the entire population of a particular generation is introduced. Also, it was combined with the basic mutation rule through a linear decreasing function. The new local mutation effectively enhanced the local search tendency of the basic DE and accelerated the convergence speed. An adaptive local search for dynamically balancing the degree of global search (GS) and local search (LS) was proposed in [25]. In this adaptive local search, if LS performs better than GS, it will increase its preference for utilization. If LS does not perform well, it will reduce its preferences for LS. The performance of the new algorithm for hybridization of the adaptive LS scheme is evaluated by using 10 benchmark problems, and the results prove the effectiveness of the algorithm. An enhanced differential evolution with random local search (named DERLS) was proposed in [26]. The advantage of using random local search in DERLS is to make a small random “jump” to a more promising area in the solution space, thus avoiding the local optimum. It is very simple, fast in calculation, and more efficient for multimode functions than classical DE. Peng et al. proposed a heterozygous differential evolution with Taguchi local search, which effectively enhances the local search performance of DE [27].

Inspired by local search methods, this paper uses the Hadamard matrix to construct the local search for DE.

## 4. Hadamard Local Search for Differential Evolution

### 4.1. Motivation

A crossover operator is a recombination operator that generates an offspring around the parents. Therefore, a local search strategy can be regarded as a moving operator [22]. In traditional DE, the binomial crossover operator (the most commonly used crossover operator) only generates and evaluates one single trial vector, which is a vertex of the hyper-rectangle defined by the mutant vector and the target vector [17]. That is to say, only one of many combinations is obtained. As a result, the search for space around parents is inadequate. On the other hand, if all vertices of the super-rectangle defined by the mutation vector and the target vector are checked, a lot of computation is needed. In this paper, a compromise method is used to construct a local search operator by using Hadamard matrix to search several vertices.

### 4.2. Local Search Based on Hadamard Matrix

A Hadamard matrix is a square matrix whose entries are either +1 or −1 and whose rows are mutually orthogonal. For example, a fourth-order Hadamard matrix (H4) is represented as follows:

(10) $$\begin{array}{c} H4=\left\{\begin{array}{cc} 1 & 1\;1\;1\\ 1 & -1\;1\;-1\\ 1 & 1\;-1\;-1\\ 1 & -1\;-1\;1 \end{array}\right). \end{array}$$

In geometric terms, this means that each pair of rows in the Hadamard matrix represents two vertical vectors [28]. Except for the first line, half of the elements in each row contain +1 and the other half −1. This feature allows us to construct a new local search strategy based on Hadamard matrix (HLS).

**Figure 1:**
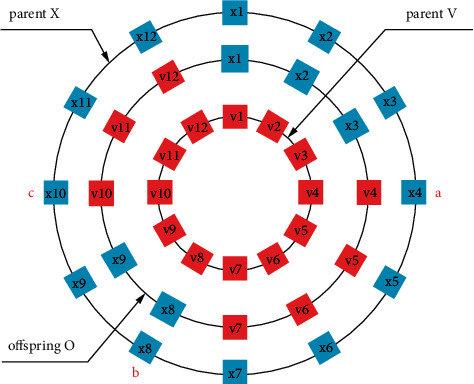
HLS example.

Based on the above characteristics of Hadamard matrix, we propose a new local search operator. We take the fourth-order Hadamard matrix H4 as an example. Since the scale of the optimization problem *d* is generally greater than 4, it is impossible to create the crossover operator directly on H4. To use H4, the d-dimensional space of the optimization problem needs to be divided into several subspaces. For example, if the dimension size of the optimization problem is 10, the interval [1, 10] should be randomly divided into four subintervals, and each interval corresponds to an element of H4.  Figure 1 shows an example of HLS.

Algorithm 1 presents the steps of HLS. With HLS, v1 and x1 will produce four offspring, among which v1 is the mutant individual of x1. Due to the characteristics of Hadamard matrix, these four offspring are four random combinations of v1 and x1. Compared with the traditional crossover operator, HLS can search more completely in local space and find better solution more easily. Therefore, HLS will improve the search performance when classical crossover cannot find a better solution.

### 4.3. New Framework of DE with HLS

There are two common ways to use the local search operator in DE algorithm. One is to replace the original crossover operator with local search operator, just as OXDE [17] did. Another method is to select an individual to perform a local search independently during evolution. In essence, for local search, the goal is to find better offspring than the target individual. Therefore, when the crossover operator can produce better offspring, there is no need for local search. In the process of evolution, the success rate of individual renewal is relatively fast in the early stage of evolution, while in the late stage of evolution, the success rate of individual renewal is very slow and even tends to zero.

**Figure 2:**
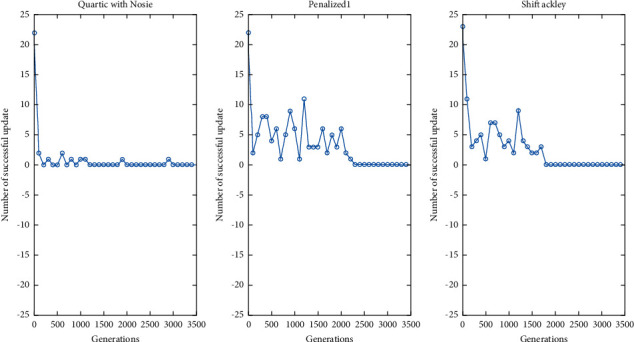
Successful update of population with generations.

In the following, we will use three representative functions: Quartic with Noise, Penalized1, and Shift Ackley, to give the convergence process. In the experiment, the population size was set to 30.  Figure 2 shows a successful single update in solving these three functions. As can be seen from Figure 2, almost all individuals cannot be successfully updated at the later stage.

To improve the success rate of DE during the later stage, a new framework of DE with HLS was proposed. HLS operator will not affect the performance of the algorithm in the early stage of evolution, but it can effectively avoid premature convergence in the late stage of evolution. The new framework is presented in Algorithm 2. To avoid consuming too much evaluation resources, the framework uses HLS with the specified probability *p* (see Algorithm 2, Step 13). During our experiments, P is set to 0.1. In addition, to make full use of the information of the evolution process, HLS uses a mutation vector to construct the local search (see Algorithm 2, Step 14).

### 4.4. Computational Complexity

For DE with HLS, computational complexity is determined by the number of times the three operators of DE and HLS are executed. Also, its execution time is proportional to the search space dimension. Consequently, DE with HLS has a worst case time complexity on the order of, where is size of population, is the maximum iteration number, and P is the user-defined probability to execute HLS. It is easy to deduce that the time complexity of DE with HLS is. In [9], the time complexity of DE is O(D × NP × GMAX). Therefore, the time complexity of DE with HLS is the same as that of DE.

## 5. Experimental Study

### 5.1. Test Suit

In our experiments, twenty widely used test functions [29, 30] are used to evaluate DEHLS. These functions include 7 unimodal functions (f1∼f7), 6 multimodal functions (f8∼f13) and 7 shifted functions (f14∼f20). The information of each function is listed in Table 1.

### 5.2. Experimental Setting

In this paper, three sets of experiments were conducted. The first set of experiments combined the HLS with four classical operators of DE algorithm to verify the validity of the HLS. The second set of experiments analysed the influence of the size growth of Hadamard matrix. The third set of experiments tested the performance of jDE [14] with HLS and compared it with four other state-of-the-art DE variants (jDE, Sade, ODE, and OXDE).

In all experiments, the dimension size (*D*) of the test problem is set to 30, the termination criterion is *D∗*10000, and 30 independent runs were conducted. In the first set of experiments, the parameters F and CR are set to 0.9, and the parameter NP is set to NP = *D*. In the third set of experiments, the parameters of the four compared algorithms are set according to the original literature.

### 5.3. Quality of the HLS

In this section, four classical schemes of DE, namely, DE/rand/1, DE/best/1, DE/rand-to-best/1, and DE/current-to-best/1, are used in the experiment to evaluate the quality of HLS. To distinguish them, these four diagrams are, respectively, named DE1, DE2, DE3, and DE4. The HLS operator has been integrated into each of the four schemes above, under the names DE1HLS, DE2HLS, DE3HLS, and DE4HLS. To guarantee the fairness of the experiment, the same parameters are defined for all the algorithms.

**Figure 3:**
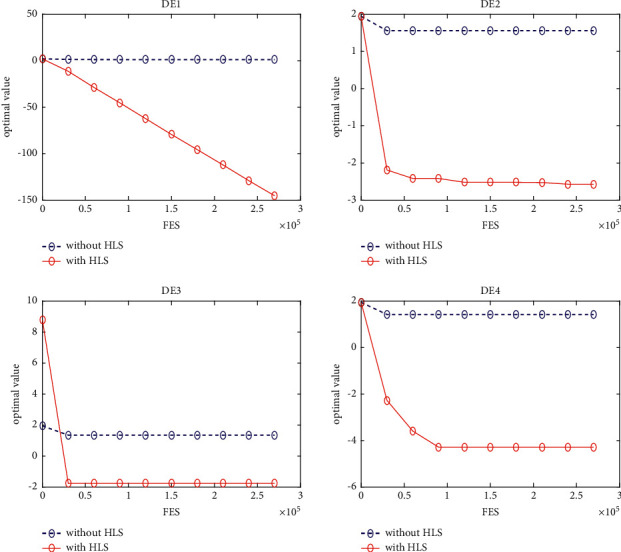
f4 convergence curves.

**Figure 4:**
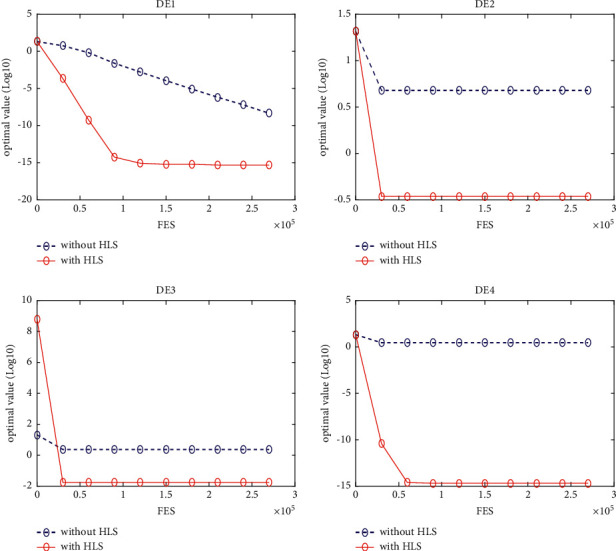
f10 convergence curves.

**Figure 5:**
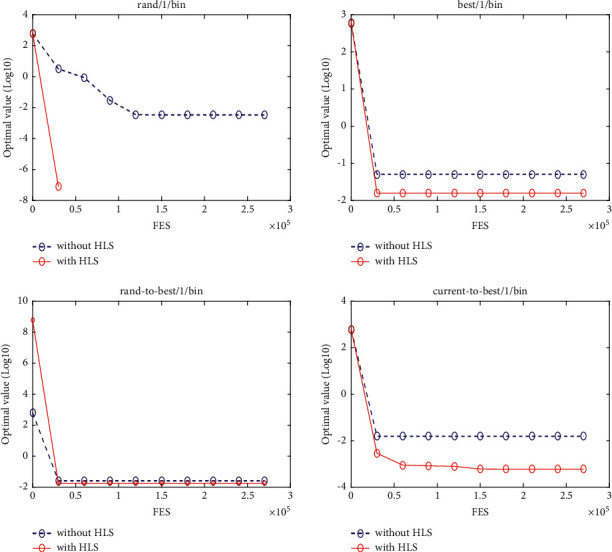
f11 convergence curves.

**Figure 6:**
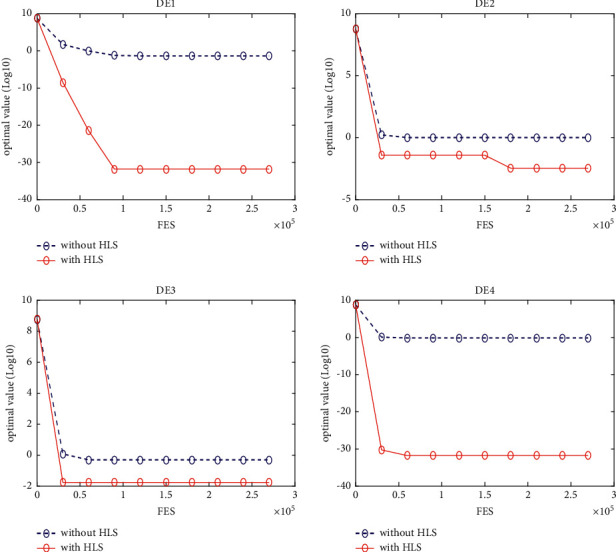
f12 convergence curves.

**Figure 7:**
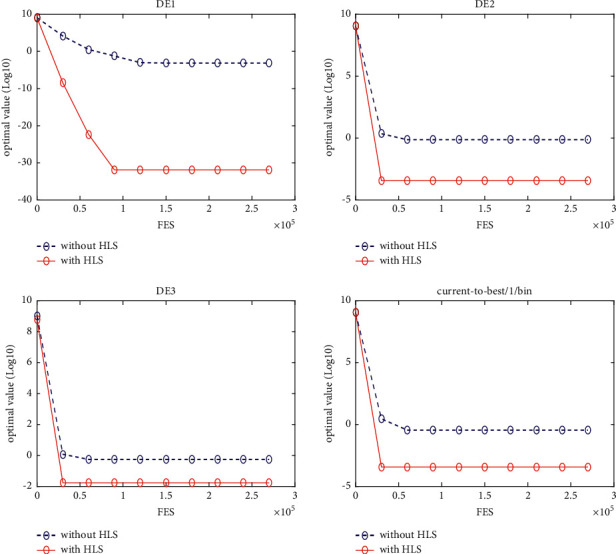
f13 convergence curves.

**Figure 8:**
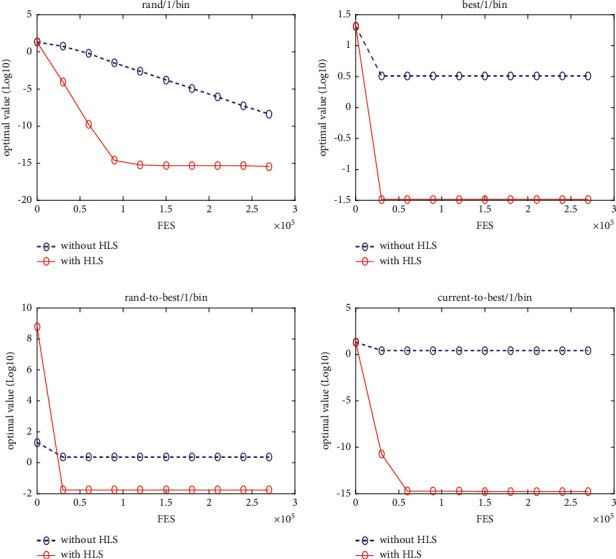
f18 convergence curves.

**Figure 9:**
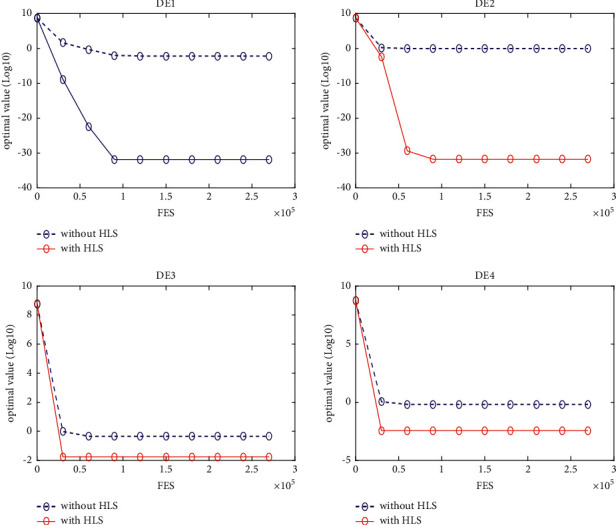
f19 convergence curves.

**Figure 10:**
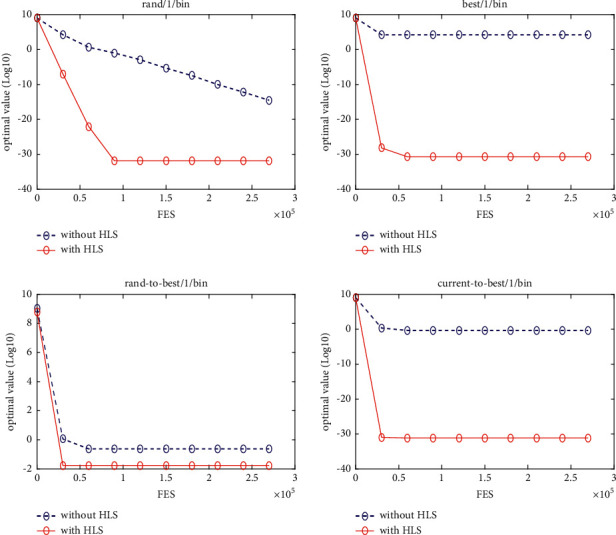
f20 convergence curves.

Table 2 presents the results of the experiment. “Average error” and “standard error” represent, respectively, the average value of error of the function and the standard deviation obtained by all the algorithms. The results of the Wilcoxon rank sum test are marked “-,” “+,” and “≈” in the table to indicate that the performance of DE without HLS is lower, better, and similar to that of DE with HLS. In addition, Figures 3–10 show the evolutionary processes of the two competitors.

From the results of Table 2, we can see that the HLS can greatly improve the performance of the four classic DE schemes. For the 20 benchmark problems, the number of functions with better results from the four DE schemes with HLS than the DE schemes was 16, 15, 13, and 13, respectively. This improvement suggests that HLS promotes the performance of the majority of test functions. Also, the number of functions with worse results from the four DE schemes with HLS than the DE schemes was 3, 4, 7, and 7, respectively. These functions are mainly unimodal functions. One of the reasons is that the solutions of the unimodal functions are easy to obtain, but HLS has increased the number of evaluations.

Therefore, we concluded that HLS can effectively improve the performance of DE, especially with multimode and shifted/offset functions.

### 5.4. Effect of the Size of Hadamard Matrix Dimension

In this section, the effect of dimension size of Hadamard matrix was analysed. Since the dimension size of the matrix is a multiple of 2 or 4, to reduce the computing burden, the dimension sizes of Hadamard matrices 4, 8, and 16 are used for experiments (written as HLS-4, HLS-8, and HLS-16). On the other hand, as can be seen from the analysis in Section 4.3, DE1HLS (DE/rand/1 + HLS) is the best one among the four schemes. Thus, DE1HLS is used in the experiment.

Table 3 summarizes the results of the experiment, in which “†“ represents the best solution for these three solutions. The statistical results are in the last row of the table.

As can be seen from the results in Table 3, when the Hadamard matrix dimension is 4, the optimal solutions of 16 problems are better than the other two dimension sizes (8 and 16).  Table 4 further gives the average ranking of the Hadamard matrices in three different dimensions (based on the Friedman test). The best average ranking is HLS_4. Thus, the following experiment will use HLS_4.

### 5.5. Implementation in jDE

In this section, the proposed HLS is implemented in jDE [14], which is a very powerful state-of-the-art DE variant. jDEHLS is compared with four state-of-the-art DE variants (jDE, SaDE, ODE, and OXDE). The experimental results and Wilcoxon's rank sum test are summarized in Table 5.

Compared with jDE, jDEHLS is superior to jDE on 10 functions and similar to jDE on 10 functions. Compared with SaDE, jDEHLS is superior to SaDE on 10 functions, but inferior to SaDE on 3 functions and similar to SaDE on 7 functions. Compared with ODE, jDEHLS is superior to ODE on 13 functions, but inferior to ODE on 1 function and similar to ODE on 6 functions. Compared with OXDE, jDEHLS is superior to OXDE on 17 functions, but inferior to OXDE on 1 function and similar to OXDE on 2 functions.

In short, HLS can improve the performance of jDE. On 20 test functions, the performance of jDEHLS is the best one among the five methods.

To judge whether the results of the five methods differ in a statistically significant way, a nonparametric statistical test called Friedman test is conducted. The test results are presented in Table 6. As shown in Table 6, the average ranking values for these five algorithms can be sorted in the following order: jDEHLS, jDE, ODE, SaDE, and OXDE.

In addition, the multiproblem Wilcoxon's test was conducted to check the behaviour of the six algorithms. The results are summarized in Table 6. We can find that *R*+ values are higher than R- in all cases, This suggests that jDEHLS is markedly superior to OXDE, SaDE, ODE, and jDE.

Figures 11–18 show the convergence process of the five algorithms on eight representative test functions. As illustrated in this eight graphs, jDEHLS converges faster than other algorithms.

**Figure 19:**
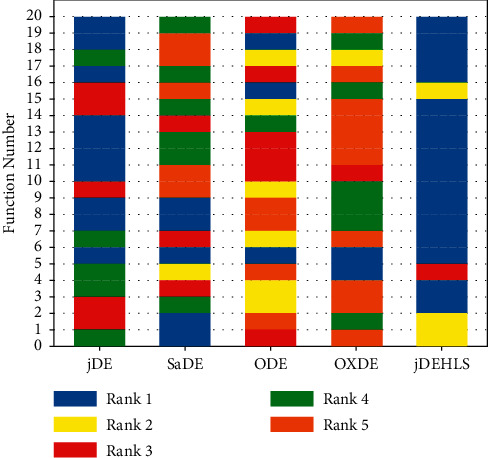
Ranking of five algorithms for 20 test functions.

Figure 19 shows the ranking of the numbers of optimal solutions achieved by each algorithm. Obviously, jDEHLS is also the first ranking.

## 6. Conclusion

This paper presents a new local search operator based on Hadamard matrix, which is called HLS. HLS searches the subspace defined by two randomly selected individuals. HLS can improve the probability of finding a better solution in a specified space by constructing multiple offspring. This is very beneficial to promote the balance between exploration and exploitation. Implementation of four classical DE algorithms and one DE variant, jDE, demonstrates the effectiveness of HLS. In future work, a parameter adaption mechanism for HLS is expected to be developed. In addition, using HLS for large-scale optimization problems will be considered. Finally, the proposed HLS operator may be used to tackle some complex real-world optimization problems.

The source code of DEHLS is available at https://github.com/gitdxg110/DEHLS_v1.

## Figures and Tables

**Figure 11:**
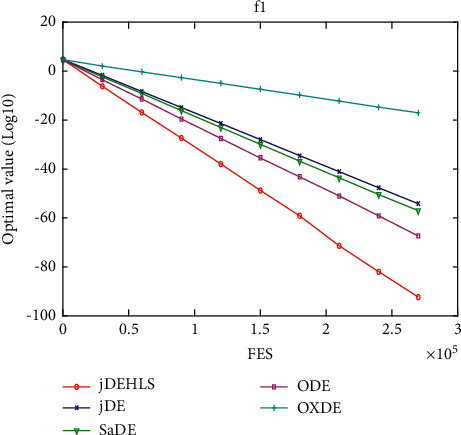
f1 convergence curves.

**Figure 12:**
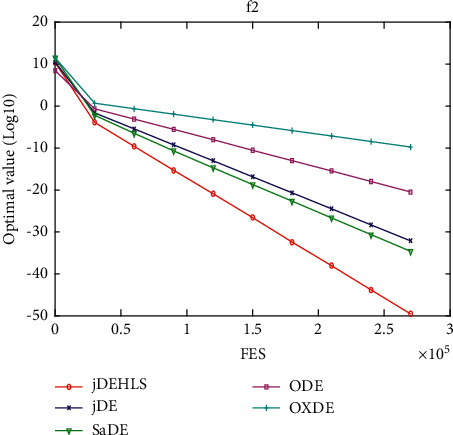
f2 convergence curves.

**Figure 13:**
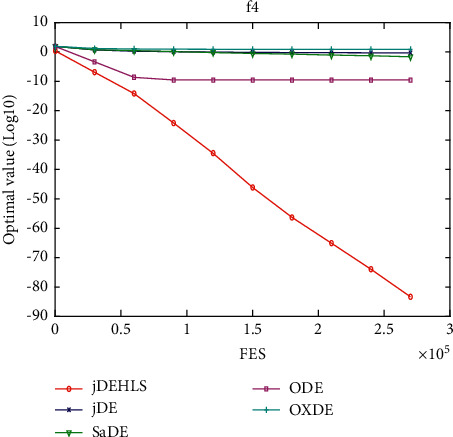
f4 convergence curves.

**Figure 14:**
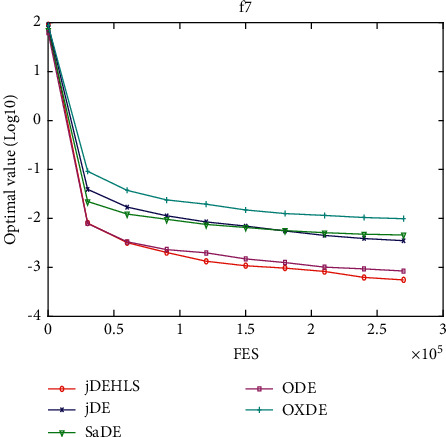
f7 convergence curves.

**Figure 15:**
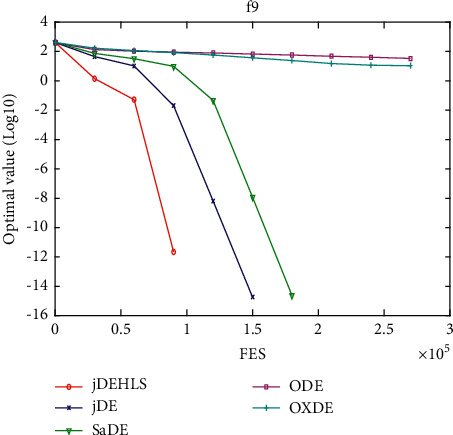
f9 convergence curves.

**Figure 16:**
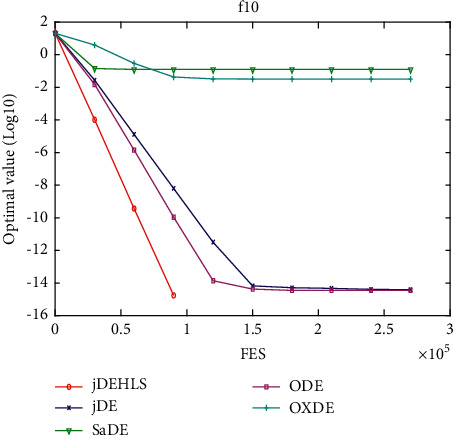
f10 convergence curves.

**Figure 17:**
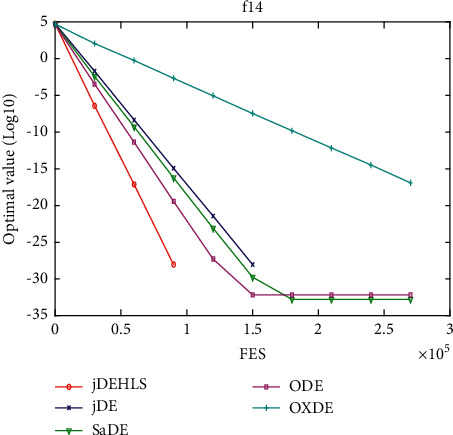
f14 convergence curves.

**Figure 18:**
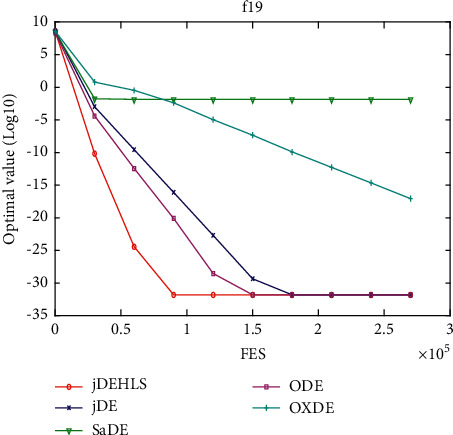
f19 convergence curves.

**Algorithm 1:**
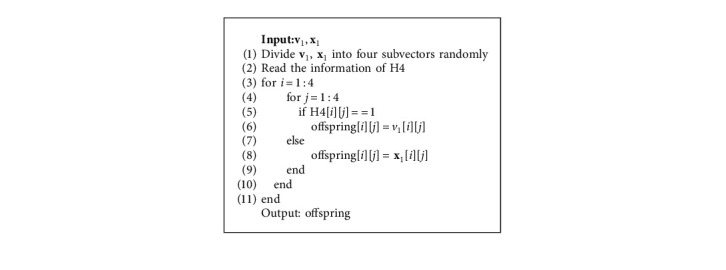
Algorithm 1 The steps of HLS.

**Algorithm 2:**
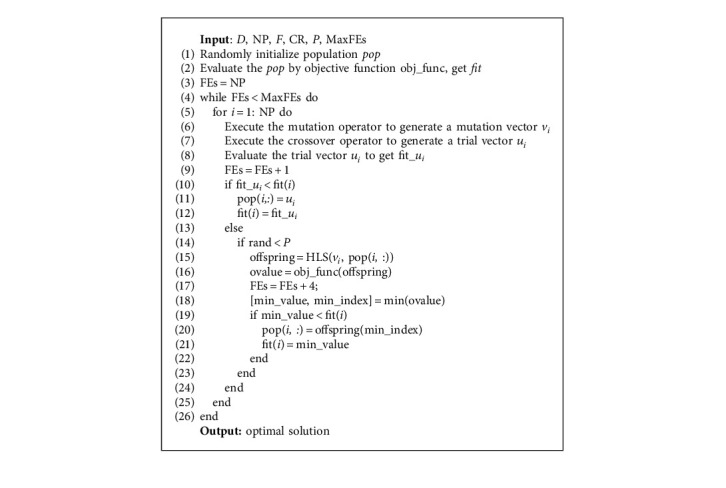
New framework of DE with HLS.

**Table 1:** Test suit.

Type	Function	Name	Dimension	*F* (*x* ^*∗*^)	Range
Unimodal functions	f1	Sphere	30	0	[−100, 100]
	f2	Schwefel 2.22	30	0	[−10, 10]
	f3	Schwefel 1.2	30	0	[−100, 100]
	f4	Schwefel 2.21	30	0	[−100, 100]
	f5	Rosenbrock	30	0	[−30, 30]
	f6	Step	30	0	[−100, 100]
	f7	Quartic with Noise	30	0	[−1.28, 1.28]

Multimodal functions	f8	Schwefel 2.26	30	−12569.5	[−500, 500]
	f9	Rastrigin	30	0	[−5.12, 5.12]
	f10	Ackley	30	0	[−32, 32]
	f11	Griewank	30	0	[−600, 600]
	f12	Penalized1	30	0	[−50, 50]
	f13	Penalized2	30	0	[−50, 50]

Shifted functions	f14	Shift sphere	30	0	[−100, 100]
	f15	Shift schwefel1.2	30	0	[−100, 100]
	f16	Shift schwefel1.2 with noise	30	0	[−100, 100]
	f17	Shift Griewank	30	0	[−600, 600]
	f18	Shift Ackley	30	0	[−32, 32]
	f19	Shift penalized1	30	0	[−50, 50]
	f20	Shift penalized2	30	0	[−50, 50]

**Table 2:** Experimental results of four classical operators of DE with HLS for all test functions and comparison without HLS.

Fun	DE/rand/1/bin	DE/rand/1/bin + HLS	DE/best/1/bin	DE/best/1/bin + HLS	DE/rand-to-best/1/bin	DE/rand-to-best/1/bin + HLS	DE/current-to-best/1/bin	DE/current-to-best/1/bin + HLS
	Mean error ± Std error	Mean error ± Std error	Mean error ± Std error	Mean error ± Std error	Mean error ± Std error	Mean err ± Std err	Mean err ± Std err	Mean err ± Std err
*f1*	3.47*e − *18 ± 7.66*e − *18−	1.12*E − *112 ± 6.03*E − *112	1.94*e − *12 ± 1.06*e − *11-	2.94*E − *20 ± 1.61*E − *19	3.41*e − *106 ± 1.85*e − *105+	1.70*E − *49 ± 9.31*E − *49	4.90*e − *108 ± 1.89*e − *107+	2.90*E − *17 ± 1.59*E − *16
*f2*	6.46*e − *11 ± 9.73*e − *11−	2.30*E − *58 ± 1.22*E − *57	1.90*e − *04 ± 1.04*e − *03≈	7.53*E − *04 ± 4.09*E − *03	1.83*e − *59 ± 7.68*e − *59+	1.36*E − *06 ± 7.47*E − *06	3.55*e − *61 ± 1.27*e − *60+	3.96*E − *12 ± 2.17*E − *11
*f3*	3.46*e − *02 ± 3.26*e − *02+	1.65*E + *00 ± 2.73*E + *00	1.55*e − *21 ± 4.76*e − *21+	2.10*E − *15 ± 7.15*E − *15	2.86*e − *24 ± 7.23*e − *24+	3.93*E − *17 ± 6.83*E − *17	6.53*e − *25 ± 1.50*e − *24+	2.39*E + *00 ± 1.31*E + *01
*f4*	1.53*e + *01 ± 7.28*e + *00−	1.73*E − *165 ± 0.00*E + *00	3.60*e + *01 ± 7.31*e + *00−	2.70*E − *03 ± 1.45*E − *02	2.23*e + *01 ± 5.68*e + *00−	3.72*E − *175 ± 0.00*E + *00	2.63*e + *01 ± 7.30*e + *00−	5.02*E − *05 ± 2.12*E − *04
*f5*	8.83*e + *00 ± 3.21*e + *00≈	1.19*E + *01 ± 1.38*E + *01	9.30*e − *01 ± 1.71*e + *00+	2.27*E + *01 ± 1.16*E + *01	1.20*e + *00 ± 1.86*e + *00+	2.55*E + *01 ± 8.66*E + *00	1.46*e + *00 ± 1.95*e + *00+	2.27*E + *01 ± 1.16*E + *01
*f6*	2.67*e − *01 ± 7.85*e − *01−	0.00*E + *00 ± 0.00*E + *00	2.27*e + *02 ± 5.80*e + *02−	0.00*E + *00 ± 0.00*E + *00	3.03*e + *01 ± 7.46*e + *01−	0.00*E + *00 ± 0.00*E + *00	1.85*e + *01 ± 2.45*e + *01−	0.00*E + *00 ± 0.00*E + *00
*f7*	1.63*e − *02 ± 5.98*e − *03−	3.54*E − *03 ± 4.00*E − *03	1.11*e − *02 ± 7.48*e − *03-	2.30*E − *03 ± 4.55*E − *03	8.50*e − *03 ± 4.20*e − *03−	1.70*E − *03 ± 3.10*E − *03	8.40*e − *03 ± 4.20*e − *03−	1.30*E − *03 ± 1.36*E − *03
*f8*	1.32*e + *04 ± 2.47*e + *02−	1.26*E + *04 ± 0.00*E + *00	1.55*e + *04 ± 6.80*e + *02−	1.38*E + *04 ± 1.77*E + *03	1.49*e + *04 ± 5.66*e + *02−	1.32*E + *04 ± 1.29*E + *03	1.54*e + *04 ± 6.62*e + *02−	1.30*E + *04 ± 1.13*E + *03
*f9*	2.41*e + *01 ± 7.72*e + *00−	0.00*E + *00 ± 0.00*E + *00	6.60*e + *01 ± 2.03*e + *01−	1.49*E + *01 ± 2.40*E + *01	3.66*e + *01 ± 8.98*e + *00−	2.78*E + *00 ± 8.54*E + *00	4.09*e + *01 ± 1.12*e + *01−	6.41*E + *00 ± 1.29*E + *01
*f10*	3.68*e − *10 ± 3.02*e − *10−	4.74*E − *16 ± 1.23*E − *15	4.78*e + *00 ± 2.44*e + *00−	3.46*E − *01 ± 1.51*E + *00	2.34*e + *00 ± 1.35*e + *00−	3.04*E − *14 ± 1.59*E − *13	2.78*e + *00 ± 2.04*e + *00−	2.01*E − *15 ± 2.02*E − *15
*f11*	3.37*e − *03 ± 8.55*e − *03−	0.00*E + *00 ± 0.00*E + *00	5.09*e − *02 ± 7.59*e − *02−	1.58*E − *02 ± 6.72*E − *02	2.55*e − *02 ± 3.08*e − *02−	3.11*E − *03 ± 1.08*E − *02	1.55*e − *02 ± 1.95*e − *02-	5.75*E − *04 ± 2.21*E − *03
*f12*	4.53*e − *02 ± 2.29*e − *01−	1.57*E − *32 ± 1.91*E − *34	1.01*e + *00 ± 2.15*e + *00−	3.46*E − *03 ± 1.89*E − *02	5.00*e − *01 ± 8.89*e − *01−	1.73*E − *02 ± 7.74*E − *02	7.25*e − *01 ± 1.00*e + *00−	1.68*E − *32 ± 2.47*E − *33
*f13*	7.32*e − *04 ± 2.79*e − *03−	1.35*E − *32 ± 5.57*E − *48	7.44*e − *01 ± 1.70*e + *00−	3.66*E − *04 ± 2.01*E − *03	5.65*e − *01 ± 1.49*e + *00−	4.01*E − *32 ± 8.12*E − *32	3.61*e − *01 ± 9.67*e − *01−	3.66*E − *04 ± 2.01*E − *03
*f14*	1.65*e − *18 ± 4.20*e − *18−	0.00*E + *00 ± 0.00*E + *00	8.77*e − *22 ± 4.80*e − *21+	1.62*E + *01 ± 8.89*E + *01	5.07*e − *30 ± 9.73*e − *30+	4.80*E − *05 ± 2.63*E − *04	3.57*e − *30 ± 2.69*e − *30+	4.16*E − *17 ± 2.27*E − *16
*f15*	3.73*e − *02 ± 3.54*e − *02+	1.16*E + *00 ± 1.75*E + *00	5.48*e − *22 ± 1.45*e − *21+	1.05*E − *15 ± 1.88*E − *15	5.30*e − *24 ± 2.06*e − *23+	1.12*E − *06 ± 6.14*E − *06	8.67*e − *25 ± 2.10*e − *24+	7.27*E − *17 ± 1.80*E − *16
*f16*	3.73*e − *02 ± 4.47*e − *02+	2.76*E − *01 ± 5.10*E − *01	9.76*e − *03 ± 4.36*e − *02−	1.42*E − *05 ± 4.99*E − *05	3.02*e − *07 ± 1.17*e − *06+	3.75*E + *00 ± 2.05*E + *01	2.75*e − *10 ± 1.34*e − *09+	8.68*E − *07 ± 4.75*E − *06
*f17*	1.81*e − *03 ± 4.92*e − *03−	0.00*E + *00 ± 0.00*E + *00	4.13*e − *02 ± 4.73*e − *02−	7.75*E − *03 ± 4.24*E − *02	1.65*e − *02 ± 1.65*e − *02−	2.47*E − *04 ± 1.35*E − *03	3.13*e − *02 ± 3.18*e − *02−	0.00*E + *00 ± 0.00*E + *00
*f18*	3.13*e − *10 ± 2.45*e − *10−	3.55*E − *16 ± 1.08*E − *15	3.25*e + *00 ± 1.95*e + *00−	1.78*E − *03 ± 9.72*E − *03	2.31*e + *00 ± 1.19*e + *00−	1.18*E − *15 ± 1.94*E − *15	2.62*e + *00 ± 9.67*e − *01−	1.66*E − *15 ± 1.80*E − *15
*f19*	6.91*e − *03 ± 3.79*e − *02−	1.57*E − *32 ± 1.77*E − *34	9.70*e − *01 ± 1.87*e + *00−	1.71*E − *32 ± 3.20*E − *33	4.53*e − *01 ± 6.95*e − *01−	1.66*E − *32 ± 1.91*E − *33	6.54*e − *01 ± 9.23*e − *01−	3.46*E − *03 ± 1.89*E − *02
*f20*	1.19*e − *17 ± 4.41*e − *17−	1.35*E − *32 ± 5.57*E − *48	1.73*e + *04 ± 9.47*e + *04−	1.96*E − *31 ± 4.78*E − *31	2.38*e − *01 ± 5.94*e − *01−	6.13*E − *32 ± 2.62*E − *31	4.67*e − *01 ± 1.63*e + *00−	6.79*E − *32 ± 2.55*E − *31

*w/b/s*	16/3/1		15/4/1		13/7/0		13/7/0	

**Table 3:** Experimental results of HLS with three sizes of Hadamard matrix.

Fun	DE/rand/1/bin + HLS_4	DE/rand/1/bin + HLS_8	DE/rand/1/bin + HLS_16
*f1*	1.12*E − *112 ± 6.03*E − *112†	7.60*E − *84 ± 4.16*E − *83	3.12*E − *59 ± 1.63*E − *58
*f2*	2.30*E − *58 ± 1.22*E − *57†	2.81*E − *45 ± 1.07*E − *44	1.15*E − *30 ± 6.27*E − *30
*f3*	1.65*E + *00 ± 2.73*E + *00†	1.61*E + *01 ± 1.58*E + *01	1.95*E + *02 ± 2.89*E + *02
*f4*	1.73*E − *165 ± 0.00*E + *00†	6.10*E − *129 ± 3.31*E − *128	1.58*E − *88 ± 7.30*E − *88
*f5*	1.19*E + *01 ± 1.38*E + *01	1.11*E + *01 ± 1.39*E + *01†	1.29*E + *01 ± 1.41*E + *01
*f6*	0.00*E + *00 ± 0.00*E + *00†	0.00*E + *00 ± 0.00*E + *00†	0.00*E + *00 ± 0.00*E + *00†
*f7*	3.54*E − *03 ± 4.00*E − *03	3.50*E − *03 ± 3.33*E − *03†	4.63*E − *03 ± 4.28*E − *03
*f8*	1.26*E + *04 ± 0.00*E + *00+	1.26*E + *04 ± 0.00*E + *00†	1.26*E + *04 ± 0.00*E + *00†
*f9*	0.00*E + *00 ± 0.00*E + *00†	0.00*E + *00 ± 0.00*E + *00†	0.00*E + *00 ± 0.00*E + *00†
*f10*	4.74*E − *16 ± 1.23*E − *15†	4.74*E − *16 ± 1.23*E − *15†	1.07*E − *15 ± 1.66*E − *15
*f11*	0.00*E + *00 ± 0.00*E + *00†	0.00*E + *00 ± 0.00*E + *00†	0.00*E + *00 ± 0.00*E + *00†
*f12*	1.57*E − *32 ± 1.91*E − *34	1.57*E − *32 ± 5.57*E − *48†	1.57*E − *32 ± 5.57*E − *48†
*f13*	1.35*E − *32 ± 5.57*E − *48	1.35*E − *32 ± 5.57*E − *48†	1.35*E − *32 ± 5.57*E − *48†
*f14*	0.00*E + *00 ± 0.00*E + *00†	0.00*E + *00 ± 0.00*E + *00†	0.00*E + *00 ± 0.00*E + *00†
*f15*	1.16*E + *00 ± 1.75*E + *00†	1.13*E + *01 ± 1.05*E + *01	1.61*E + *02 ± 1.52*E + *02
*f16*	2.76*E − *01 ± 5.10*E − *01†	2.88*E + *00 ± 3.635*E + *00	4.42*E + *01 ± 4.02*E + *01
*f17*	0.00*E + *00 ± 0.00*E + *00†	0.00*E + *00 ± 0.00*E + *00†	0.00*E + *00 ± 0.00*E + *00†
*f18*	3.55*E − *16 ± 1.08*E − *15†	3.55*E − *16 ± 1.08*E − *15†	9.47*E − *16 ± 1.60*E − *15
*f19*	1.57*E − *32 ± 1.77*E − *34†	1.58*E − *32 ± 7.07*E − *34†	1.57*E − *32 ± 5.57*E − *48†
*f20*	1.35*E − *32 ± 5.57*E − *48†	1.35*E − *32 ± 5.57*E − *48†	1.35*E − *32 ± 5.57*E − *48†
*Best Num*	16	14	10

**Table 4:** Friedman test results.

Algorithm	Friedman value
DE/rand/1/bin + HLS_4	1.85
DE/rand/1/bin + HLS_8	1.95
DE/rand/1/bin + HLS_16	2.22

**Table 5:** Experimental results of jDE, SaDE, ODE, OXDE, and jDEHLS for all functions.

	jDE	SaDE	ODE	OXDE	jDEHLS
*f1*	1.31*e − *61 ± 2.02*e − *61-	4.10*e − *131 ± 1.54*e − *130+	1.39*e − *75 ± 6.59*e − *75-	3.67*e − *59 ± 7.32*e − *59-	2.83*E − *118 ± 1.05*E − *117
*f2*	1.91*e − *36 ± 1.75*e − *36-	2.80*e − *79 ± 5.88*e − *79+	1.21*e − *23 ± 1.29*e − *23-	4.09*e − *33 ± 3.66*e − *33-	3.92*E − *62 ± 1.13*E − *61
*f3*	2.67*e − *07 ± 4.76*e − *07-	5.66*e − *07 ± 1.25*e − *06-	4.98*e − *08 ± 6.62*e − *08-	2.91*e − *05 ± 3.39*e − *05-	6.29*E − *09 ± 1.80*E − *08
*f4*	8.05*e − *01 ± 1.72*e + *00-	2.11*e − *07 ± 1.15*e − *06-	2.89*e − *10 ± 1.58*e − *09-	7.08*e + *00 ± 4.64*e + *00-	2.15*E − *94 ± 1.18*E − *93
*f5*	9.58*e + *00 ± 9.25*e − *01-	3.14*e + *00 ± 2.61*e + *00+	2.53*e + *01 ± 8.26*e − *01-	1.20*e + *00 ± 1.86*e + *00+	4.68*E + *00 ± 1.06*E + *01
*f6*	0.00*e + *00 ± 0.00*e + *00≈	0.00*e + *00 ± 0.00*e + *00≈	0.00*e + *00 ± 0.00*e + *00≈	0.00*e + *00 ± 0.00*e + *00≈	0.00*E + *00 ± 0.00*E + *00
*f7*	3.38*e − *03 ± 8.43*e − *04-	2.60*e − *03 ± 1.10*e − *03-	7.87*e − *04 ± 2.23*e − *04-	4.08*e − *03 ± 1.59*e − *03-	4.68*E − *04 ± 3.12*E − *04
*f8*	3.82*e − *04 ± 0.00*e + *00≈	3.82*e − *04 ± 0.00*e + *00≈	6.57*e + *03 ± 5.69*e + *02-	3.94*e + *00 ± 2.16*e + *01-	3.82*E − *04 ± 0.00+00
*f9*	0.00*e + *00 ± 0.00*e + *00≈	0.00*e + *00 ± 0.00*e + *00≈	2.91*e + *01 ± 1.78*e + *01-	1.01*e + *01 ± 2.79*e + *00-	0.00*E + *00 ± 0.00*E + *00
*f10*	3.91*e − *15 ± 1.08*e − *15-	1.24*e − *01 ± 3.22*e − *01-	3.55*e − *15 ± 0.00*e + *00-	3.10*e − *02 ± 1.70*e − *01-	0.00*E + *00 ± 0.00*E + *00
*f11*	0.00*e + *00 ± 0.00*e + *00≈	2.22*e − *03 ± 4.90*e − *03-	1.15*e − *03 ± 3.02*e − *03-	1.15*e − *03 ± 3.09*e − *03-	0.00*E + *00 ± 0.00*E + *00
*f12*	1.57*e − *32 ± 5.57*e − *48≈	3.46*e − *03 ± 1.89*e − *02≈	1.58*e − *32 ± 2.63*e − *34-	1.04*e − *02 ± 3.16*e − *02-	1.57*E − *32 ± 5.57*E − *48
*f13*	1.35*e − *32 ± 5.57*e − *48≈	1.10*e − *03 ± 3.3*e − *03≈	1.37*e − *32 ± 9.00*e − *34≈	1.83*e − *03 ± 8.20*e − *03≈	1.35*E − *32 ± 5.57*E − *48
*f14*	0.00*e + *00 ± 0.00*e + *00≈	1.64*e − *33 ± 9.00*e − *33≈	6.57*e − *33 ± 3.60*e − *32≈	1.31*e − *31 ± 5.89*e − *31	0.00*E + *00 ± 0.00*E + *00
*f15*	2.07*e − *07 ± 2.95*e − *07-	3.28*e − *07 ± 5.69*e − *07-	1.03*e − *07 ± 2.14*e − *07-	1.45*e − *05 ± 1.59*e − *05-	5.6*E − *08 ± 2.93*E − *07
*f16*	3.87*e − *07 ± 4.23*e − *07-	1.66*e − *01 ± 5.26*e − *01-	1.21*e − *07 ± 1.63*e − *07+	1.15*e − *04 ± 1.95*e − *04-	1.99*E − *07 ± 1.08*E − *06
*f17*	0.00*e + *00 ± 0.00*e + *00≈	2.22*e − *03 ± 5.35*e − *03-	3.29*e − *04 ± 1.80*e − *03≈	2.55*e − *03 ± 4.51*e − *03-	0.00*E + *00 ± 0.00*E + *00
*f18*	3.67*e − *15 ± 6.49*e − *16-	3.10*e − *02 ± 1.70*e − *01-	3.55*e − *15 ± 0.00*e + *00-	3.55*e − *15 ± 0.00*e + *00-	0.00*E + *00 ± 0.00*E + *00
*f19*	1.57*e − *32 ± 5.57*e − *48≈	1.18*e − *02 ± 3.21*e − *02-	1.57*e − *32 ± 5.57*e − *48≈	1.58*e − *32 ± 3.29*e − *34≈	1.57*E − *32 ± 5.57*E − *48
*f20*	1.35*e − *32 ± 5.57*e − *48≈	3.66*e − *04 ± 2.01*e − *03≈	1.61*e − *32 ± 1.44*e − *32≈	1.10*e − *03 ± 3.35*e − *03-	1.35*E − *32 ± 5.57*E − *48
*−/+/≈*	10/0/0	10/3/7	13/1/6	16/1/3	

**Table 6:** Statistical results of jDE, SaDE, ODE, OXDE, and jDE based on the multiproblem Wilcoxon's test.

jDEHLS vs	*R*+	R-	*P* value	Α = 0.05
OXDE	182.5	27.5	0.003592	4.20
SaDE	162.5	27.5	0.005954	3.45
ODE	194.5	15.5	0.000736	3.05
jDE	174.5	16.0	0.001378	2.65

## Data Availability

The data used to support the findings of this study are included within the article.
